# Exploring the determinants of subjective well-being among the elderly in Dongguan: a qualitative comparison of migrant and local residents

**DOI:** 10.3389/fpsyg.2025.1534637

**Published:** 2025-08-14

**Authors:** Li Jia, Hang Cheng, Jinzhi Huang, Huanting Liu, Qihui Gan, Xianglei Zhu, Jin Huang, Qikang Chen, Xiao Lin, Yuxi Liu

**Affiliations:** ^1^Shunde Women and Children’s Hospital, Guangdong Medical University, Foshan, China; ^2^School of Humanities and Management, Guangdong Medical University, Dongguan, China; ^3^Tangxia Town Community Health Service Center of Dongguan City, Dongguan, China; ^4^Institute of Health Law and Policy, Guangdong Medical University, Dongguan, China

**Keywords:** subjective well-being, migrant elderly, native elderly, qualitative research, Dongguan

## Abstract

**Objectives:**

This study aims to explore the determinants of subjective well-being among elderly populations in Dongguan, one of China’s most rapidly industrializing cities, with a particular focus on differences between migrant and native elderly residents.

**Methods/analysis:**

A qualitative research design was employed. Semi-structured in-depth interviews were conducted with 38 elderly participants (26 native and 12 migrant residents) in Tangxia Town, Dongguan City. The data were analyzed using thematic analysis supported by NVivo software, and participant recruitment was conducted through purposive and snowball sampling until data saturation was reached.

**Findings:**

Five main themes influencing subjective well-being emerged: physical condition, family relationships, basic living conditions, environmental adaptation, and life security. Health and family reunification were universally regarded as critical to well-being. While both groups reported positive well-being, migrant elderly experienced more barriers in medical insurance coverage and lower engagement in social activities, contributing to relatively lower satisfaction. Native elderly, in contrast, benefited more from established social networks and local policy support.

**Novelty/improvement:**

Unlike previous studies that focus predominantly on migrant workers or treat elderly populations as a homogeneous group, this study offers a comparative lens on migrant and native elderly, revealing unique challenges faced by migrant elders in urban environments. The findings highlight the need for more inclusive social participation opportunities and portable medical insurance policies to improve the well-being of migrant elderly in rapidly urbanizing areas. This study contributes empirical evidence to inform aging-related policy development under China’s demographic transition.

## 1 Introduction

With China’s rapid demographic transition and deepening urbanization, the country has witnessed a substantial rise in both its aging population and internal migration. According to the Seventh National Population Census released in 2021, China’s floating population reached 376 million in 2020, representing a 69.73% increase over the past decade. Meanwhile, the number of people aged 60 and above reached 264 million, indicating a deepening trend of population aging (Ning, 2021). However, unlike younger migrant workers who relocate for employment or education, elderly migrants—often referred to as “wandering elderly” or “migrant elderly”—typically move to urban areas to reunite with family, care for grandchildren, or receive care from adult children (Ruan et al., 2019; Liu et al., 2014). Despite their growing numbers and social relevance, migrant elderly remain a marginalized group in scholarly discourse and policy design, often facing institutional exclusions and limited access to urban welfare systems (Liu et al., 2017; Sand and Gruber, 2018).

Subjective well-being (SWB), defined as a person’s cognitive and affective appraisal of their life satisfaction and emotional experiences (Bum et al., 2015), is a core dimension of successful and healthy aging. A growing body of literature has demonstrated that SWB is influenced by both personal and contextual factors, including physical health, family relationships, social support, financial security, and environmental conditions (Bum et al., 2015; Lee et al., 2021; Jiang and Renema, 2021). In the case of older adults, SWB is not only a psychological outcome but also a predictor of health behaviors, mental resilience, and quality of life (Zhang and Liu, 2011; Zhong et al., 2017). International studies have highlighted that migrant elderly often experience lower SWB due to cultural adaptation stress, limited local support networks, language barriers, and exclusion from welfare services (Sand and Gruber, 2018; Yang et al., 2018; Jeon et al., 2016; Jane et al., 2012). However, several critical challenges remain in existing research. First, although international literature has explored the well-being of migrant elderly in contexts such as Europe, North America, and Australia (Sand and Gruber, 2018; Bum et al., 2015; Lee et al., 2021; Jiang and Renema, 2021), studies from China tend to focus either on young migrant workers or treat the elderly as a homogeneous population. Few have examined how SWB is differentially experienced between migrant and native elderly within the same urban context, particularly in southern China’s rapidly developing regions. Second, many studies adopt quantitative approaches using national survey data, which, while valuable, may overlook the complex social and emotional realities of older migrants (Liu et al., 2017; Zhong et al., 2017; Stenlund et al., 2022). Furthermore, although physical health and economic security are recognized as strong predictors of SWB among elderly populations (Wang and Kim, 2020; Jang et al., 2020), the role of interpersonal and institutional factors—such as social activity participation, access to public services, and feelings of social exclusion—remains underexplored in the Chinese context. Migrant elderly may face unique disadvantages in these areas, including unfamiliar environments, weak social networks, and fragmented medical insurance systems (Jang and Tang, 2022; Dong et al., 2012). These factors may not only hinder their adaptation but also reinforce structural inequalities in aging outcomes.

To address these gaps, this study investigates the factors influencing SWB among both migrant and native elderly living in Dongguan, a city representative of China’s manufacturing-driven development and high-density population mobility. Specifically, the study adopts a qualitative comparative approach to explore how physical health, family relationships, social activity, environmental adaptation, and life security shape SWB across the two groups. Semi-structured interviews were conducted with 38 elderly participants (26 native and 12 migrant), and thematic analysis was employed to extract meaningful patterns of experience. This study makes three key contributions:

First, it provides one of the few comparative qualitative analyses between migrant and native elderly in a rapidly urbanizing Chinese city, thus offering fine-grained insights that are often obscured in survey-based studies.

Second, it identifies institutional and social participation gaps—such as limited medical insurance portability and lower involvement in recreational activities—as critical barriers to migrant elderly’s well-being, thus complementing the existing health- and income-centered frameworks.

Third, it proposes actionable recommendations for local aging policy, including the development of inclusive community engagement platforms and the reform of cross-regional medical insurance mechanisms.

Through these contributions, the study not only enhances theoretical understanding of aging and well-being in China’s internal migration context but also provides evidence-based guidance for creating more equitable and supportive urban environments for elderly populations.

## 2 Materials and methods

### 2.1 Study design and theoretical framework

This study employed a qualitative descriptive design using semi-structured in-depth interviews, guided by a constructivist paradigm. The research was informed by the social-ecological model of aging and theoretical perspectives on SWB, which posit that individual well-being in later life is shaped by multiple interacting levels—personal health, family relationships, community resources, and institutional support (Bum et al., 2015; Lee et al., 2021; Jiang and Renema, 2021; Zhang and Liu, 2011). The study followed the COREQ (Consolidated Criteria for Reporting Qualitative Research) guidelines.

### 2.2 Research setting and timeframe

The fieldwork was conducted in August 2022 in Tangxia Town, Dongguan City, Guangdong Province, China. Dongguan is one of China’s most rapidly industrialized cities and has experienced significant internal migration over the past two decades. Tangxia Town, in particular, has a high density of migrant and native elderly residents, making it an ideal setting for comparative study.

### 2.3 Participant selection and recruitment

Participants were recruited using purposive sampling followed by snowball sampling. Initial selection focused on capturing maximum variation in terms of migration status (migrant vs. native), gender, age group (60–74, 75+), and educational background. Inclusion criteria were: (1) aged 60 years or older, (2) permanent or temporary residents of Tangxia Town for at least 6 months, and (3) cognitively able to provide informed consent and participate in the interview.

The recruitment process began through community health centers and neighborhood committees in Tangxia. With assistance from local coordinators, we identified 6 initial participants (“seed informants”), who then referred other eligible individuals. Recruitment continued until data saturation was reached, defined as the point at which no new themes emerged from the interviews. A total of 38 participants were interviewed, comprising 26 native elderly and 12 migrant elderly.

### 2.4 Interview guide and data collection

The interview guide was developed based on an extensive literature review and discussion with experts in gerontology, public health, and qualitative research. The guide covered five major domains:

1. Personal health and functional status,
2. Family relationships and support,
3. Material conditions and financial security,
4. Social activity and community integration, and.
5. Perception of public services and environmental adaptation.

Before the interviews, participants were provided with oral and written informed consent materials. Each interview lasted 30 to 40 min and was conducted in Mandarin or Cantonese, depending on the participant’s preference, by trained researchers with backgrounds in qualitative methods and elderly care. All interviews were audio-recorded with permission and supplemented by field notes, which documented non-verbal cues such as facial expressions, posture, and tone. Interviews were conducted in private, quiet settings, either in participants’ homes or community activity rooms, to ensure comfort and confidentiality.

### 2.5 Data analysis

Audio recordings were transcribed verbatim and cross-checked by a second researcher for accuracy. All transcripts were imported into NVivo 12 for analysis. Thematic analysis was performed using Braun and Clarke’s six-phase method:

1. Familiarization with data,
2. Generating initial codes,
3. Searching for themes,
4. Reviewing themes,
5. Defining and naming themes, and.
6. Producing the report.

An inductive coding strategy was employed to allow themes to emerge from the data, while still being informed by the study’s theoretical framework. To enhance reliability, two independent coders conducted the initial coding and met regularly to resolve discrepancies and refine the codebook. Data triangulation (comparing across migrant vs. native elderly) and peer debriefing with qualitative research advisors were used to strengthen analytic validity.

### 2.6 Trustworthiness and rigor

To ensure credibility, we used member checking by summarizing key themes to a subset of participants and confirming the accuracy of interpretation. Dependability was enhanced through audit trails and detailed documentation of coding decisions. Confirmability was supported by reflexive journaling by interviewers. Transferability was addressed by providing a detailed description of the research context and participant characteristics.

### 2.7 Ethical considerations

Ethical approval was obtained from the Institutional Ethics Committee of Guangdong Medical University (Reference Number: YS2022092). Participants were informed of their right to withdraw at any time without consequences. All personal identifiers were removed during transcription, and audio files were securely stored. Confidentiality and anonymity were strictly maintained throughout the research process.

## 3 Results

All 38 invited elderly (26 non-migrants and 12 migrants) agreed to participate in the study.

### 3.1 Basic characteristics of the elderly

Of the participants in this study, 26 had been living in Dongguan since birth, and 12 were from other parts of mainland China, most of them from underdeveloped and rural areas. Table 1 demonstrates the basic characteristics of the two groups of older adults, the vast majority of whom were under 90 years of age, and the gender and age of the two groups were generally similar.

**Table 1:** Group characteristics of migrants and native elderly people (*n* = 38).

Participant ID	Migration status	Gender	Age group
N01	Native	Female	60–74
N02	Native	Female	60–74
N03	Native	Female	60–74
N04	Native	Female	60–74
N05	Native	Male	60–74
N06	Native	Female	60–74
N07	Native	Female	60–74
N08	Native	Female	60–74
N09	Native	Female	60–74
N10	Native	Male	60–74
N11	Native	Female	60–74
N12	Native	Female	60–74
N13	Native	Female	60–74
N14	Native	Female	75–90
N15	Native	Male	75–90
N16	Native	Female	75–90
N17	Native	Female	75–90
N18	Native	Female	75–90
N19	Native	Female	75–90
N20	Native	Male	75–90
N21	Native	Female	75–90
N22	Native	Female	75–90
N23	Native	Female	75–90
N24	Native	Female	75–90
N25	Native	Male	90+
N26	Native	Female	90+
M01	Migrant	Male	60–74
M02	Migrant	Female	60–74
M03	Migrant	Male	60–74
M04	Migrant	Male	60–74
M05	Migrant	Male	60–74
M06	Migrant	Female	60–74
M07	Migrant	Male	60–74
M08	Migrant	Female	60–74
M09	Migrant	Male	60–74
M10	Migrant	Female	75–90
M11	Migrant	Male	75–90
M12	Migrant	Female	90+

### 3.2 Perceived contribution to SWB

Physical condition, family composition, basic conditions, life security and environmental atmosphere were the five common factors affecting mobility and local elderly people’s SWB.

#### 3.2.1 Physical condition

Table 2 presents the key statements that illustrate participants’ perceptions of the relationship between health and SWB. Good physical condition was consistently recognized by both migrant and native elderly as a crucial contributor to their well-being. Health was commonly described as a form of personal capital that enhances one’s quality of life, while illness was viewed as a major threat that diminishes emotional satisfaction and limits daily functioning. This finding aligns with previous studies suggesting that self-rated physical health is one of the strongest predictors of SWB among elderly populations (Stenlund et al., 2022; Wang and Kim, 2020). Nearly all participants explicitly identified their physical condition as the most important factor influencing their sense of happiness and life satisfaction.

**Table 2:** Relationship between participants perceived physical condition and SWB.

Participants	Good health and a high sense of SWB	Poor health and low SWB
Native elderly	“I do not have to worry about my children, we all want to be in good health, now I just need to be in good health.”	
	“I think it’s best if I can eat, drink and walk.”	
	“Gee, I’m older now, I do not have to think about so many things, I do not have to worry about the kids and all that. So, the important thing is health, and health is money, do not you think? Now it says the most important thing is health, otherwise you are useless even if you have money.”	
		“Physical pain and mobility problems can sometimes affect my mood.”
Migrant elderly	“For everyone it’s health that’s most important, and a lot of the ones younger than me have passed away. They all started exercising when they felt they were unhealthy. Unlike me, I started exercising when I was young and have been exercising consistently for decades.”	
	“Now I do not have to worry about my children. It’s not good to think too much, but now I feel that it does not matter anymore, as long as I sleep, eat and digest well.”	
		“It bothers me when I’m not well. Bad health can affect my mood.”

#### 3.2.2 Family relations

There are many reasons elderly move to a new city, most of them to be with family, partly to care for grandchildren, partly to hope that their children will take care of them. In some cases, family harmony takes precedence over the needs of the elderly themselves. The conflict between the elderly and their children in the way of life and behavior will affect the family relationship, and then lead to depression in the elderly (Jang et al., 2020; Jang and Tang, 2022; Dong et al., 2012). During the interviews, most of the elderly said that despite some minor conflicts in their lives, family relationships were generally harmonious. Because of their good family relationships, they rarely argue with the family and do not have many worries. Nonetheless, this perspective may not reflect the experience of elderly individuals whose family ties are weak, or those who feel emotionally excluded even within co-residence arrangements.

“My family is very well connected. I live with my son and daughter-in-law and twin grandchildren. I enjoy living with them and my granddaughter and grandson often call and care for me.” (Native elderly).

“The days of being poor are over. I now have children, food and a house to live in, as well as good health. Living with my daughter-in-law I do not have to do anything.” (Native elderly).

“My grandson would come and play with me on weekends or during winter and summer vacations. I also like to take care of my grandchildren because when they come over, I will not be so bored and the house will be a little more lively.” (Native elderly).

“I live with my children now, and they are very filial. I take care of my grandchildren and my children take care of me when I get sick.” (Migrant elderly).

“My son is a businessman and I moved here with him. What I am most happy about is that my children are married and have grandchildren. My grandchildren have graduated from university and are now working.” (Migrant elderly).

“My daughter-in-law likes to be shady, and I like people who are straightforward, not shady.”(Migrant elderly).

#### 3.2.3 Basic conditions

The results of the assessment of the perception of basic conditions, including material needs and social activities, were generally the same for the migrant and local elderly.

##### 3.2.3.1 Material needs

All respondents expressed material needs being met. In the material needs, the satisfaction of material base and financial subsidies affect the SWB of the elderly. The migrant elderly and native elderly have a relatively solid material foundation, and the government gives some financial subsidies from time to time. This makes the elderly group more satisfied.

“I’m happy to have my own house and live in it and eat. I have money to eat. The most basic thing for elderly is to have money to eat. Elderly people get money every month when they are old.” (Native elderly).

“Because the production team and the village committee give out money every year, I have enough money to spend without spending the children’s money. I used to have to farm to be rich, now I do not have to work to be rich. I will plant vegetables when I am free, these vegetables are enough for me to eat, I do not have to go to the market to buy vegetables.” (Native elderly).

“I have a monthly pension of 4,000(CNY) and I’m quite happy with that. Recently there will be some additional pension payments, and with more pension, my life will be a bit better.” (Migrant elderly).

“All aspects of life are better now than before. My son gives me 3,000(CNY) a month to live on and I do not have to worry about money.” (Migrant elderly).

##### 3.2.3.2 Social activities

Although living in the same area, there is a slight gap between migrant and native residents in terms of social activities. All the native elderly answered that they had a variety of social activities, including fitness exercises, community friendships, recreational activities and group travel, which effectively relieved their stress and prevented possible loneliness. The vast majority of migrant elderly also expressed a desire to be actively involved in social activities. However, although some migrant elderly say they are very interested in social activities, they do not take practical actions, which may increase their loneliness.

“A few of us elderly have been to many places with tour groups. Happy with our current life, nothing else to pursue.” (Native elderly).

“I usually play mahjong for a long time with one yuan or two yuan as a bet, just for entertainment and to kill time. When I have nothing to do, I chat while playing mahjong. Gradually, I feel better.” (Native elderly).

“My usual entertainment is playing on my phone, some neighbors or fellow villagers I know will occasionally ask me to play with them, and the neighbors are all okay.” (Migrant elderly).

“When I was in my hometown, I would play mahjong if there was nothing going on. I do not know anyone here, and when I want to play mahjong I cannot find anyone. I have to be at home every day and it’s quite boring.” (Migrant elderly).

#### 3.2.4 Environment

It’s divided into three parts, including Infrastructure supporting, facilities and impact of COVID-19. Migrants and natives generally have the same perception of these three aspects.

##### 3.2.4.1 Infrastructure supporting

All migrants agree that the environment in Dongguan City is better than where they live before, Dongguan City has a good climate, fresh air and order. The vast majority of native elderly have the same opinion, but they want urban planning to take the needs of seniors more into account. Although better urban infrastructure is perceived positively, the extent to which it translates into higher well-being may vary depending on individual adaptability and digital literacy, particularly in contexts of rapid urbanization.

“Life and other places are also very important. It seems that our neighboring village is also very satisfied. It is very clean, and all walks of life around it is also very good. These are quite good. These places are very good, and the air is also very good Where we live, the air is good.” (Native elderly).

“Life here is very good, you say Guangdong, Shenzhen, Guangzhou, and Dongguan are all the best. You all know that well, I do not know if others are comfortable or not, but I’m very comfortable living here.” (Native elderly).

“I’m satisfied with the life here, it’s very good, I do not worry about it here, the security is good, and the environment is okay. No Like when we first came here, at that time motorcycle is banned, society was improving, so we should be grateful.” (Migrant elderly).

##### 3.2.4.2 Facilities

Immigrant and native elders have similar views on infrastructure. Most of the elderly expressed their satisfaction with the city in terms of infrastructure and transportation services, but also expressed difficulties in adapting to the development of industrialization. Lack of skills to operate computers and mobile phones, they preferred to pay with cash than with mobile phones, which affects their integration into urban life.

“It’s very convenient to travel. I usually buy groceries in the supermarkets around the community. Now everything is very good, you can buy medicine, you can buy breakfast.” (Native elderly).

“Old people do not know how to use mobile phones, your young people It’s the most convenient, you do not need to bring money to buy things. You can also spend money in the future. But the biggest problem for us elderly people is that we are not used to it, we are old.” (Native elderly).

“I do not know how to use WeChat to buy I will be deceived by others. Now many things and mobile phones are deceiving people. I do not listen to some calls. We listen to the familiar calls, and we do not listen to the unfamiliar ones.” (Native elderly).

“I think it’s very convenient, A lot of necessary infrastructure has been provided, which fully meets my needs.” (Migrant elderly).

“Everything is good now, just this computer, the computer cannot be used, the mobile phone cannot be used, and the children bought me an operating system Books, but that book is not reliable. Sometimes some messages will be sent in the group, and I do not know how to use a mobile phone, so it’s very inconvenient.” (Migrant elderly).

##### 3.2.4.3 Impact of COVID-19

The impact of COVID-19 on the elderly is relatively serious, which largely limits the travel of the elderly, and the burden of safety issues is heavy when traveling. Although they hope to travel more places while they can still walk age, but they do not want the trouble that may bring for their children and themselves.

“Now what do we old people want? I am dozens of years old. I cook and drag the ground to make these things. I want to go out and travel. A long time ago, if it wasn’t for the epidemic, I do not know how to travel for dozens of days a year, and the epidemic is not good, so I went on a trip before.” (Native elderly).

“We all feel that the epidemic has a great impact on us, and we are worried about where we go, and we are afraid of where we will go. I cannot come back due to the epidemic, which makes my children worry.” (Native elderly).

“I do not really want to go anywhere now. Because of the epidemic, I live in Tangxia. Before the epidemic, I wanted to go everywhere. Because I’m leaving now, if you get older, you will not be able to leave, old people are like this.” (Migrant elderly).

#### 3.2.5 Living security

Life security includes both medical and material insurance, and there were both similarities and differences in the perceptions of migrants and native elderly regarding these two areas.

##### 3.2.5.1 Material insurance

There is no big difference between migrants and native elderly. Almost all the elderly expressed their satisfaction with the security of material life. These guarantees mainly include government benefits, subsidies for the elderly, and holiday condolences.

“The ones in the community are very good to the elderly. They give us food every day. Water, oil, and rice. Every month.” (Native elderly).

“For the elderly and poor, the Mid-Autumn Festival There are moon cakes and a little money, and they will be available during Chinese New Year, and there will be benefits for the elderly, and the elderly will have benefits.” (Migrant elderly).

##### 3.2.5.2 Medical insurance

Migrant and native elderly hold opposing views. Most native elderly are satisfied with the security of medical treatment, but most migrants feel that there is a lack of security from medical treatment. Migrants expect more support in medical insurance, reimbursement anywhere in China, and further increase the proportion of medical insurance reimbursement.

“The community takes good care of us elderly. The community doctors will come to check us up from time to time and come to our home to provide some services.” (Native elderly).

“The medical insurance claims are very high. For example, if I buy more than 400, I only need to pay more than 100. The money for taking medicine every month is much less.” (Native elderly).

“The medical care is still not up to date, the medical care is not up to date, like what disease do I have, the reimbursement of the medical insurance is too low. Outpatient clinics in other places cannot be reported at all.” (Migrant elderly).

“I came from Guangxi, so what about the medical insurance when I come to Guangdong? Reimbursement for outpatient clinics is not available nationwide now, and you have to pay in cash. I want to make invoices, a series of things. Reimbursement is troublesome.” (Migrant elderly).

“A few years ago, I had to have a leg operation. After the examination at the hospital here, I was asked to pay 40,000 to 50,000 yuan. I was taken aback. I calculated my pension and thought it was not worth it, so I went back to my hometown for surgery. The hospital here has no way to reimburse. The hometown can be reimbursed, saving a lot of money.” (Migrant elderly).

Our study investigated the SWB of migrant and local elderly adults living in one of China’s most rapidly industrializing regions. Both groups of elderly adults shared similar perceptions of SWB as a balance between positive and negative emotions, which has also been found in other studies (Ximena et al., 2017; Zhang and Zhang, 2017; Elena et al., 2017).

However, this health-centric interpretation may downplay other psychosocial factors such as resilience, community support, or purpose in life that also influence well-being (Procházka and Bočková, 2024; Tuan et al., 2024; Hail et al., 2024). Elderly who claimed good health and family harmony had relatively positive evaluations of SWB (Table 3). Our study found that health is the basis for participating in all social works. Similar to previous studies, good physical condition is one of the most important prerequisites for maintaining SWB in older adults (Carlson et al., 1998; Amit and Howard, 2010). Influenced by Confucian culture, most Chinese, especially the elderly attaches great importance to family, and good family relationship are the source of happiness. Contrary to international migration, the main purpose of domestic elderly migration is to reunite with their families, which is also the greatest need of the elderly in China, thus partially offsetting the lack of medical insurance (Nishita and Browne, 2013; Xiong and Han, 2022).

**Table 3:** Perceptions of SWB among migrant and local elderly.

Factors	Subdivisions	Perceived effects of factors	
		Migrant elderly	Native elderly
Physical conditions		++	++
Family relationship		++	++
Basic conditions	Material needs	+	+
	Social activities	+/−	+
Environment	Infrastructure	+	+
	Supporting facilities	+/−	+/−
	Impact of COVID-19	−	−
Life insurance	Material insurance	+	+
	Medical insurance	−	+

+, positive; −, negative; ±, neutral.

## 4 Discussion

Social activity plays a critical and well-documented role in shaping the SWB of elderly individuals, exerting predominantly positive effects across various cultural contexts (Wang et al., 2020). In the current study, a clear disparity was observed between migrant and native elderly in terms of their engagement in social activities. Native elderly participants reported a higher frequency and diversity of participation—ranging from community fitness, group travel, to recreational games—whereas migrant elderly, although expressing interest in similar activities, demonstrated considerably lower levels of actual engagement. This discrepancy reflects not only differing levels of social integration, but also structural and psychological barriers faced by migrant elderly, such as unfamiliarity with the new social environment, limited peer networks, and perceived exclusion. The absence of regular social interaction often leads to weakened social ties, which in turn exacerbates feelings of isolation—creating a self-reinforcing cycle that further restricts their participation in community and leisure life (Liu et al., 2019; Shen, 2014; Shen and Yeatts, 2013). Empirical studies have consistently found that older migrants in urban China who lack social engagement are significantly more vulnerable to psychological distress, including symptoms of depression and loneliness (Tonui et al., 2022). In contrast, native elderly—having resided long-term in their communities—tend to possess stronger social networks and cultural familiarity, which facilitates more active and sustained involvement in group activities. These forms of engagement not only mitigate emotional stress but also enhance a sense of belonging, purpose, and overall well-being in later life (Feng et al., 2019).

Migrants and native elderly were generally satisfied with the city’s supporting facilities, although they still complained of some dissatisfaction. A common problem is that they all expressed the difficulty of operating computers and mobile phones, and not making payments through the use of cell phones between the two groups of elderly, reflecting the incompatibility of the elderly with the development of industrialization. Similar studies have also expressed the difficulties of the elderly in accepting and using new technologies (Li et al., 2021), which may lead to their confusion and even a sense of loneliness and isolation (Murugan et al., 2022).

In terms of medical insurance, migrants and native elderly adults have different perceptions, with most immigrant elderly adults reporting a lack of adequate medical insurance. Our research found that the immigrant elderly did not fully benefit from medical insurance, among other things, in their new place of immigration and complained about the cumbersome process of health insurance reimbursement in their new city, choosing to pay out-of-pocket in most cases to avoid the hassle, and the low reimbursement rates, which hindered immigrant older adults’ access to appropriate health services. The medical insurance of native residents is superior to that of migrant elderly in terms of procedure and reimbursement ratio (Zhang et al., 2017). Elderly have negative perceptions of convenience and accessibility of insurance. They feel unfair and unhappy when they realize the disparity between local elderly and their access to healthcare. The COVID-19 had a negative impact on SWB on both immigrant and native elderly, as it had significantly affected the travel of the elderly, and had a certain impact on the mental health of the elderly, which has also been mentioned in previous studies (Su et al., 2022; Gustafsson et al., 2022; Dhakal et al., 2022).

While migrant elderly in our study widely emphasized health as a key factor in their SWB, it is important to interpret this finding in light of existing migration-health hypotheses. The “Healthy Migrant Effect” suggests that individuals who choose to migrate are often in better physical condition than their non-migrating peers, which may partially explain the generally positive health status reported by some migrant participants. At the same time, the “Salmon Bias” hypothesis proposes that migrants in declining health may return to their hometowns, potentially leading to an overrepresentation of relatively healthier migrants in the host city. Although our study did not explicitly test these mechanisms, acknowledging them provides a more nuanced understanding of the health-related responses and points to the need for future studies to integrate migration trajectories and return patterns into analysis.

This study conducted qualitative research on the potential influencing factors of SWB of Chinese migrant and native elderly. We found that physical condition, family relationship, basic conditions, environmental atmosphere, and life insurance had different effects on improving the SWB of the elderly, which can help increase the understanding of the common determinants of the SWB of migrants and native elderly, provide evidence and reference for the government to formulate healthier aging policies, and improve the SWB of migrants and native elderly at the individual, societal, and national levels.

Some new insights emerged from this study, such as insufficient social activities of migrant elderly, which has a great impact on their SWB. Consistent with this is previous research finding that lack of social interaction among migrant elderly limits their mental health (Schoenmakers et al., 2017; Liu et al., 2017). Secondly, inadequate access to medical insurance also affects the welfare benefits of migrant elderly, thus affecting the SWB. Therefore, addressing discriminatory attitudes toward migrant elderly and providing appropriate social support is imperative. There is need to create conditions for social activities of migrant elderly, provide community planning services, and promote positive interactions between migrant and native elderly. The reform of medical insurance policy should take into account the migrant elderly, improve the medical insurance procedures in different places, and further increase the reimbursement ratio of medical insurance.

## 5 Conclusion

This study explored the determinants of SWB among migrant and native elderly in Dongguan, a rapidly urbanizing city in China. Through comparative qualitative analysis, we identified five major domains that influence elderly well-being: physical health, family relationships, material conditions, social participation, and institutional support. Among these, physical health and family reunification emerged as the most critical and universally recognized contributors to well-being. Migrant elderly exhibited generally lower levels of SWB, largely due to limited access to social activities and medical insurance coverage, while native elderly benefited from stronger community ties and localized welfare entitlements. Additionally, the COVID-19 pandemic negatively impacted both groups by restricting mobility and increasing emotional stress.

Theoretically, this study contributes to the literature by applying the social-ecological model of aging to examine how individual, interpersonal, and institutional factors interact to shape SWB in later life. It expands existing research by offering a comparative, migration-sensitive lens in a non-Western urban context, thereby enriching our understanding of health and happiness in aging populations. Moreover, this study reveals how structural exclusions—such as the fragmentation of medical insurance—may counteract family-based migration benefits and exacerbate well-being disparities. We acknowledge several limitations. First, the study was confined to a single city and may not capture regional diversity across China. Second, all participants were recruited through community networks, potentially excluding the most isolated elderly. Third, while the qualitative approach allows for rich thematic insight, future mixed-methods or longitudinal studies are needed to quantify these dynamics over time and across broader samples. Fourth, although we collected general background information on participants’ migration history, such as place of origin and duration of residence, these variables were not included in the thematic analysis. Future studies could incorporate migration trajectories more explicitly to further understand how the timing, distance, and duration of migration may shape SWB among elderly migrants.

In summary, this research contributes new knowledge by demonstrating how migration status intersects with institutional access and social engagement to shape SWB in urban China. The findings highlight the need for more inclusive aging policies, particularly in expanding social activity opportunities and ensuring portable, equitable medical insurance systems for migrant elderly. These insights can inform health and social policy in other aging societies experiencing rapid internal migration.

## Data Availability

The raw data supporting the conclusions of this article will be made available by the authors, without undue reservation.
